# Effects of interpreting a dynamic geometric cue as gaze on attention allocation

**DOI:** 10.1167/jov.23.8.8

**Published:** 2023-08-07

**Authors:** Inka Schmitz, Wolfgang Einhäuser

**Affiliations:** 1 Physics of Cognition Group, Institute of Physics, Chemnitz University of Technology, Chemnitz, Germany; 2 Physics of Cognition Group, Institute of Physics, Chemnitz University of Technology, Chemnitz, Germany

**Keywords:** cueing, gaze, motion cue, social

## Abstract

Gaze is a powerful cue for directing attention. We investigate the interpretation of an abstract figure as gaze modulates its efficacy as an attentional cue. In each trial, two vertical lines on a central disk moved to one side (left or right). Independent of this “feature-cued” side, a target (black disk) subsequently appeared on one side. After 300 trials (phase 1), participants watched a video of a human avatar walking away. For one group, the avatar wore a helmet that visually matched the central disk and looked at black disks to either side. The other group's video was unrelated to the cueing task. After another 300 trials (phase 2), videos were swapped between groups; 300 further trials (phase 3) followed. In all phases, participants responded more quickly for targets appearing on the feature-cued side. There was a significant interaction between group and phase for reaction times: In phase 3, the group who had just watched the avatar with the helmet had a reduced advantage to the feature-cued side. Hence, interpreting the disk as a turning head seen from behind counteracts the cueing by the motion of the disk. This suggests that the mere perceptual interpretation of an abstract stimulus as gaze yields social cueing effects.

## Introduction

In social interactions, the direction of another person's gaze often provides important cues for directing one's own attention—for example, when observing sources of danger or looking in the direction of a possible path. People often actively use the possibility of pointing at someone or something with their gaze. In doing so, a gaze movement often triggers a gaze-following movement, or a covert shift of attention (for a review, see Frischen, Bayliss, & Tipper, 2007). A very simple form of gaze following, presumably based on a preference for faces in combination with motion cues, is already evident in newborns (Farroni, Massaccesi, Pividori, & Johnson, 2004). Gaze as an intuitive cue to guide another individual's attention has recently also become of interest to applied fields such as robotics and human–machine interface research, as human–robot collaboration can be facilitated by gaze-based interactions (e.g., Foster, By, Rickert, & Knoll, 2006), and a huge variety of anthropomorphic gaze displays have been in use (for an overview, see Admoni & Scassellati, 2017). In this context, it is critical to know when the gaze-likeness of an abstract display suffices to induce gaze-following behavior in humans.

When the eyes of a cartoon face look toward a peripheral target, responses to the target are faster than when the eyes look straight ahead or to the opposite side (Friesen & Kingstone, 1998). The effects are in many respects similar to those of predictive cues in Posner's (1980) endogenous cueing paradigm, even though the gaze-based cue does not contain information about the position of the target (i.e., it is non-predictive in the experimental context). A large number of studies have shown the robust effect of such gaze cueing, although the size of the effect depends on task type and stimulus properties (for a review, see Dalmaso, Castelli, & Galfano, 2020; for a meta-analysis, see McKay, Grainger, Coundouris, Skorich, Phillips, & Henry, 2021). As an example, schematic faces (like those of Friesen & Kingstone, 1998) typically lead to smaller effect sizes than real faces (e.g., Talipski, Bell, Goodhew, Dawel, & Edwards, 2021).

Based on a comprehensive review on the role of attention in the selection of social interaction, Capozzi and Ristic (2018) argued for three distinct routes of processing: perception, interpretation, and evaluation. Although classical gaze cueing with isolated face images belongs mainly to the perceptual route, these authors also highlighted links to interpretive (mind–gaze associations) and socio-evaluative (weighting social information sources) routes, especially for more complex stimuli. For example, participants respond to a target more efficiently when another person appears to look at it intentionally than when the other person's looking behavior appears to be motivated socially (Colombatto, Chen, & Scholl, 2020). Colombatto et al. (2020) showed the observed persons from the back, and participants inferred gaze orientation from their head orientation. Employing gaze-based cues without visibility of the gaze source's eyes is also possible when the face is directed toward the observer—for example, a gaze source wearing sunglasses (Böckler, van der Wel, & Welsh, 2015). This is also consistent with neurophysiological results in nonhuman primates, suggesting that eye, head, and body orientations are integrated to estimate gaze direction, prioritizing (in case of conflict) the eye over the other effectors (Perrett, Hietanen, Oram, Benson, & Rolls, 1992), and with specialized circuits for processing gaze orientation in humans (Marquardt, Ramezanpour, Dicke, & Thier, 2017). Here, we, in turn, ask whether even for an isolated geometric stimulus its interpretation as head (seen from the back) impacts how much it directs the observer's attention.

To distinguish the biological relevance of gaze cues from merely overtrained symbols, arrows are frequently used as control. There is evidence that gaze elicits more robust Posner-like cueing effects than arrows (e.g., requiring less feature congruence with the target), even if the eyes are reduced to merely two circles (Ristic, Wright, & Kingstone, 2007). Similarly, arrows produce less interference during eye movements to an incongruent target than incongruent gaze (Ricciardelli, Bricolo, Aglioti, & Chelazzi, 2002). This dissociation between gaze and arrows was corroborated by the case of a patient whose gaze following was impaired but she showed unimpaired following to arrows (Akiyama, et al., 2006). Despite such differences between gaze and arrows, neither arrows nor gaze orientations can be fully ignored (Galfano, et al., 2012), suggesting that both can trigger automatic attentional shifts. Moreover, there is evidence that, for both gaze and arrows, involuntary attentional orientation is modulated by volitional cue use, depending on individual differences (Tipples, 2008). Regarding the interaction of such volitional and involuntary mechanisms, experiments in humans (Hill, et al., 2010) and nonhuman primates (Marciniak, Dicke, & Thier, 2015) suggest that there may be two phases of gaze-related attention allocation: an initial reflexive behavior followed by a more flexible gaze under more cognitive control, in which the reflexive mechanism can be voluntarily inhibited. In sum, although arrows and gaze seem to produce similar attentional effects, their effects may have different origins (Chacón-Candia, et al., 2023). Gaze and eyes may be processed automatically mainly because of their high biological relevance; arrows, because they represent a very well-learned symbol. It is therefore plausible that there are shared processes related to directional communication (gaze and arrow) and, in addition, processes that are specific to social perception (gaze only). Irrespective of whether these mechanisms and processes are clearly separable for the case of gaze, we here consider the question of whether the cognitive interpretation of a simple stimulus as gaze yields attentional effects that are similar to gaze following for actual gaze stimuli.

In the present study, we used a dynamic cue that is initially perceived as unrelated to gaze. Through video presentation, during the experiment we signaled to one group of participants that the cue in fact resembles a head as seen from behind. As the implied movement direction of the head was opposite the geometric motion of the cue, we hypothesized that interpreting a cue as gaze orientation will immediately result in gaze-based cueing effects that counteract effects resulting from the appearance and motion of the cue. That is, the interpretation of an abstract, geometric cue as a social signal may affect attentional guidance, even if it conflicts with the direction implied by the motion of the cue.

Motion is an important signal component that allows humans to infer social states. Several point-light walker studies have presented observers with the motion of a few light points attached to a human's heads and joints. From these motion signals, participants can decode information about gender, identity, and emotions, among other features (e.g., Barclay, Cutting, & Kozlowski, 1978; Troje, Westhoff, & Lavrov, 2005; Walk & Homan, 1984). Biological motion has walking direction as a directed social component, and indeed it has been shown that light-spot movements, even if not consciously perceived as foot movements, induced reaction time advantages in walking direction in a Posner-like task. No significant advantage was found for similar light-spot movements that did not correspond to biological foot movements (Wang, Yang, Shi, & Jiang, 2014). Movement also plays an important role in the context of the so-called Watching Eyes effects (Conty, George, & Hietanen, 2016), where the display of human and abstract gaze stimuli influences time perception, but in this context the mere movement of a stripe pattern showed no effects (Burra & Kerzel, 2021). In turn, a participant's cognitive model for how a visually ambiguous motion cue is coupled to actual movement can profoundly impact the coupling between movement and motion perception, and the cognitive model in these cases can be induced and switched rapidly (Veto, Uhlig, Troje, & Einhäuser, 2018). Here, we aim to exploit such rapid adjustments of cognitive models about motion stimuli to give our abstract geometric stimulus a social component—the interpretation as another person's gaze direction.

**Figure 1.**
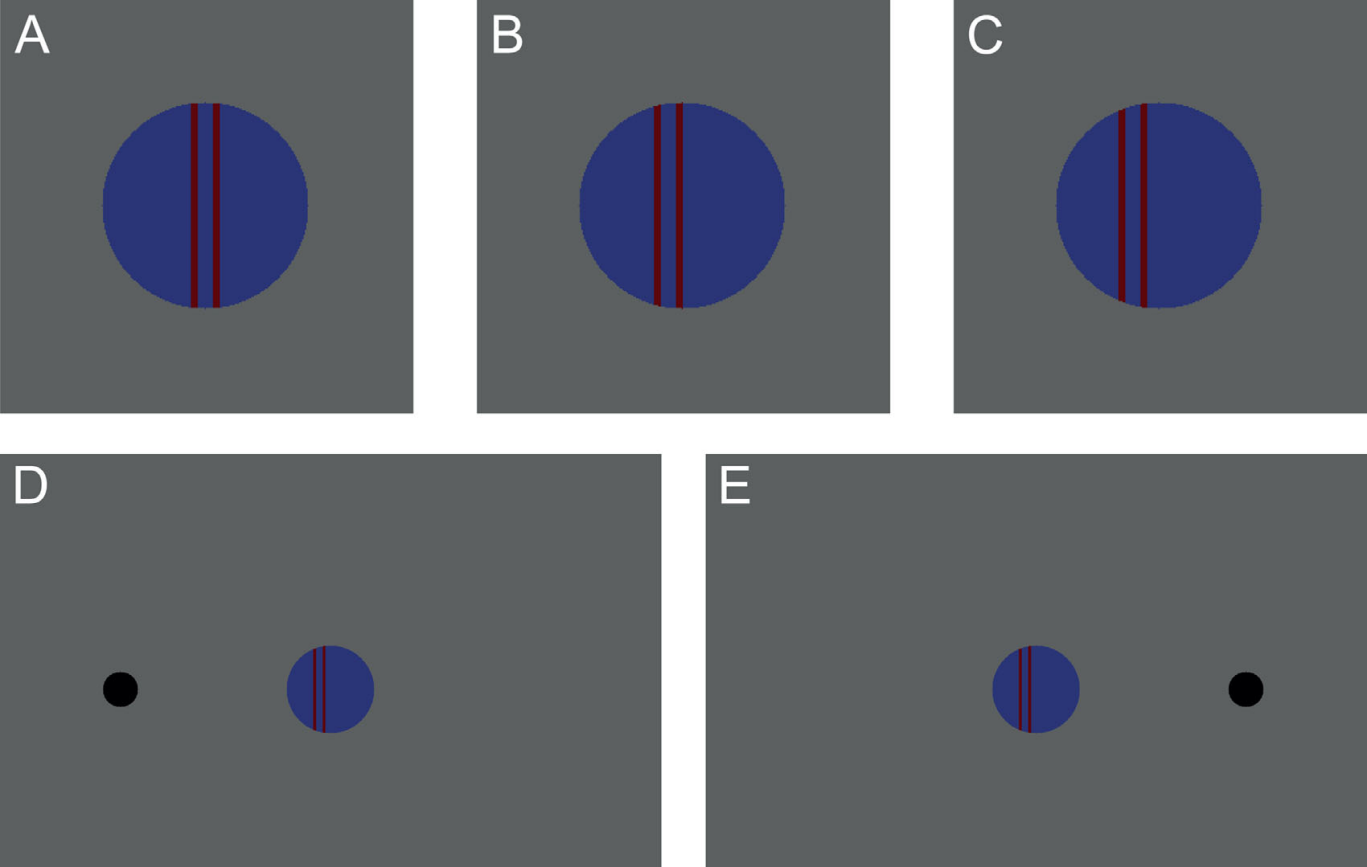
Illustration of the stimulus. (**A**) Start position of lines. (**B**, **C**) Lines moving to the left side. (**D**) Target on the feature-cued side. (**E**) Target on the gaze-cued side.

In the current study, we investigated whether changing the perceptual interpretation of the cue can influence its effect on the detection of lateralized targets, even when the cue and target remain physically unchanged. We used a blue disk with two red vertical lines on it as the cueing stimulus (Figure 1). In each trial, one side was cued by moving the lines horizontally from the center to one side. The task of the participants was to react via button press when a smaller black target disk appeared on the left or right side of the cueing disk. Importantly, as is typical for gaze cuing tasks and different from a typical Posner paradigm, the cue contained no information; that is, the target appeared with equal probability on either side of the cue and the side was independent of the motion direction of the cue.

**Figure 2.**
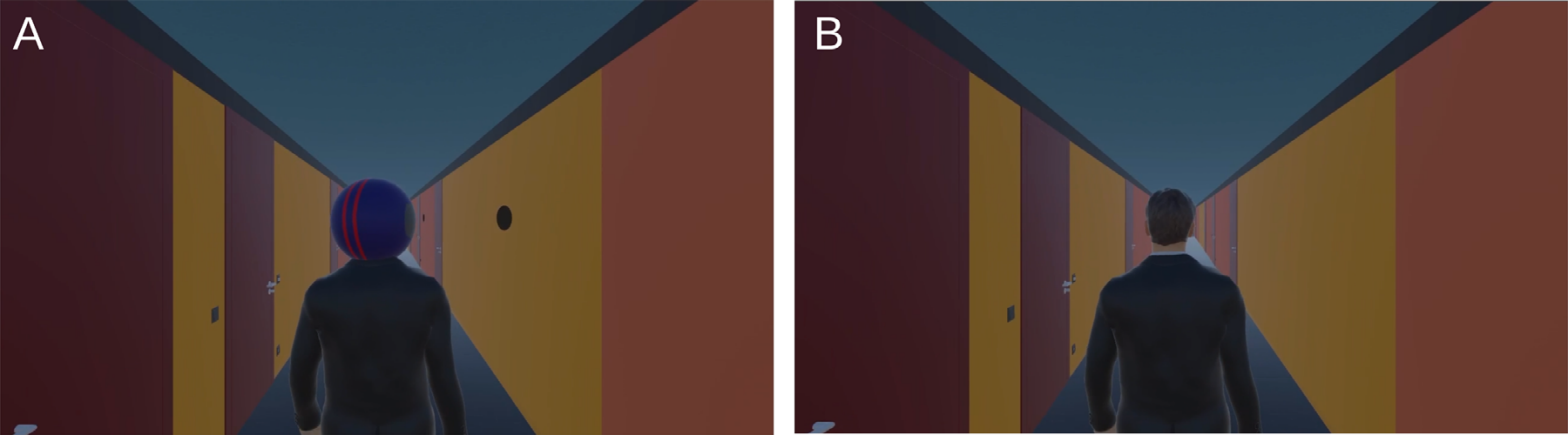
Video sequences. (**A**) Induction video. (**B**) Control video. Videos are available as Supplementary Materials and in high resolution at https://osf.io/q5t6y/. As there was an uneven number of targets in the induction video (five) and to reduce the repetitiveness of the videos, video versions that were mirror reversed at the vertical midline were also used; every other participant of each group started with the mirror-reversed version and then saw the original in block 2 and the mirror-reversed version in block 3 of the respective experiment phase. The order was reversed for the other participants (block 1 and 3 original, block 2 mirrored).

To induce an alternative interpretation of the cue, we showed a short video sequence that introduced the cueing disk as the back of a walking person's head who is looking at black disks similar to a target on the side walls of a corridor (Figure 2A). Before the presentation of the video sequence, we expected a reaction time and accuracy advantage for the feature-cued side (i.e., the side the lines on the cue move toward). We hypothesized that the participant's interpretation of the cue induced by the video presentation would lead to a benefit in accuracy and reaction time for the gaze-cued side (i.e., the side the lines on the cue move away from, which is the side the head points to), thus reducing or even inverting the feature-cueing effect. Details of the hypotheses, which were preregistered together with the analysis strategy prior to commencing the study (https://doi.org/10.17605/OSF.IO/8NSGK), are provided in the Methods section.

## Methods

### Participants

On the basis of an a priori power analysis, we included 21 participants in each of two groups (*N* = 42 in total; 16 males; age range, 19–36 years, *M* = 24, *SD* = 3.82). For calculation of the sample size, the power of our follow-up *t*-tests was crucial (see below). Assuming a large effect (*d* = 0.8), an alpha level of 0.05, and a power (1-beta) of 80%, for the directed hypothesis (reaction time [RT] difference between gaze-cued and feature-cued, decreasing) we determined that a group size of 21 per group (total sample size of *N* = 42) was required.

Each participant completed 900 trials, 300 in each phase of the experiment. For one participant we had to exclude the first 100 trials from analysis due to a misunderstanding of the instruction (reacting to the cued side rather than to the target), which could be rectified after the first block. The participants were recruited among the members of the Chemnitz University of Technology community via a dedicated mailing list and the circle of acquaintances of the experimenters. The local ethics commission (Ethikkommission, TU Chemnitz) reviewed and approved all procedures (Case No. #101549112).

### Stimuli and setup

Videos were generated using Unity3D (Unity Technologies, San Francisco, CA); all other stimuli were generated in MATLAB (MathWorks, Natick, MA). All stimuli were presented using MATLAB with the Psychophysics Toolbox (Brainard, 1997; Pelli, 1997) on a VIEWPixx monitor (size, 523 × 300 mm; resolution, 1920 × 1080 pixels; refresh rate, 120 Hz; distance from observer, 57 cm; VPixx Technologies, Saint-Bruno-de-Montarville, QC, Canada). The experiment was conducted in a testing chamber, with no light source other the monitor. We measured the gaze positions at a 1000-Hz sampling rate with the EyeLink 1000 camera/infrared-system (SR Research, Kanata, ON, Canada). The participant’s head was positioned in a chin and forehead rest for a stable viewing position in front of the screen and the eye-tracking camera. The participants’ responses were captured with the left and right button of the RESPONSEPixx Button Box (VPixx Technologies).

In all phases of the experiment, we used a cue that should initially be perceived as an abstract configuration: two vertical lines that in each trial moved either to the left or to the right inside the cueing disk (Figure 1A). To match its average position in the induction video (see below), the disk was placed 1.4 degrees of visual angle (dva) below the center of the screen.

At the beginning of each trial, the cue appeared and started to move after a 0.5- to 0.6-second delay (drawn from a uniform distribution) in one direction. This motion lasted 0.125 seconds and spanned 0.42 dva to either the left or right (Figures 1A–1C). Concurrent with the last frame of the movement, a target disk (small black disk) appeared 10 dva either left or right of the central disk (Figures 1D and 1E).

To induce an interpretation of the blue disk as a head from behind (i.e., to induce the cognitive model of a gaze cue in the participants), the participants watched a video (induction video) in which the cueing disk was presented as the back of a walking person's head looking at black disks similar to a target on the side walls of a corridor (Figure 2A). For comparison purposes, we created a visually similar control video in which it was not suggested that the cue was the back of a person's head (Figure 2B). The videos lasted 47 seconds, and the first trial after each video started 1 second after the end of the video. For details on the dynamics of the avatar's head movements, see the Appendix.

### Procedure

The experiment was divided into one three phases with three blocks of 100 trials each. Twenty-one participants were assigned to each group (group 1 and group 2). The assignment was done by alternating assignments of the participants to either group. As there was no particular order of recruitment, this equaled a random assignment procedure. The first phase (baseline phase) was identical for both groups. At the beginning of each block (i.e., thrice per experimental phase) of the second and third phase, video sequences of 47 seconds were shown. In phase 2, half of the participants (group 1) watched the induction video. The other half (group 2) watched the control video. In phase 3, the groups reversed their roles compared to phase 2; that is, group 2 participants watched the video inducing the perception of the cue as the back of a head, and group 1 participants watched the control video.

The participant's task was to respond as quickly as possible to the target by pressing a corresponding button (left button for target on the left, right button for target on the right). In the first phase, observers were not instructed regarding the meaning of the cue. Here, we expected a slight advantage in accuracy and reaction times if the target appeared on the side the lines were moving to (feature-cued side). For the second phase, we hypothesized that the video would lead group 1 to perceive the lines on the disk as a gaze cue directed opposite the feature-cued side (gaze-cued side). Consequently, we hypothesized that, after the video presentation, there would be a benefit in accuracy and reaction times for the gaze-cued side for group 1 that was not present for group 2. Regarding the third phase, we hypothesized that this would obliterate the difference between the groups or even slightly reverse it as compared to phase 2.

### Analyses

#### Reaction time and accuracy

We measured the RT from presenting the target to pressing the response button. For this analysis, we only used correct trials (i.e., trials where the correct button was pressed). For each block, we computed for each observer the difference between the median reaction time in feature-cued trials (lines on central disk moved toward the target) and gaze-cued trials (lines on the central disk moved away from the target as they would if the central disk were the back of a head looking at the target location), and we averaged these medians over the three blocks of each phase. This RT difference (RT_diff_) served as a dependent variable. The main reason for using medians is that RT distributions are usually leptokurtic (heavy-tailed), such that the median is a more robust estimate than the mean. In addition, using medians requires fewer assumptions about the distribution and provides insensitivity to outliers. RT_diff_ was then subjected to a 2 × 3 analysis of variance (ANOVA) with the between-subject factor group and the within-subject factor experiment phase. The use of medians does not violate the assumptions for ANOVA as a parametric test because the ANOVA as used herein makes no assumption about the underlying distribution per participant/phase; normality is only required for the distribution of these medians across participants and phases. Statistically, our hypotheses translate into the expectation of an interaction between the factors, which then should be followed up by independent sample *t*-tests, where a group difference would be expected for the second experiment phase (see preregistration at https://doi.org/10.17605/OSF.IO/8NSGK). Because accuracy (percent correct responses) was too close to the ceiling (97.02% across all conditions), we (in line with our preregistration) decided against analyzing accuracy.

#### Eye tracking

To verify that the induction video indeed induced gaze-following behavior, we analyzed horizontal eye position during the presentation. We aligned each participant's horizontal eye-position data to the avatar's head motion in the video, with *t* = 0 being the peak head orientation toward the target (cf. Figure 2a). We then averaged over the 15 instances (3 blocks × 5 targets) of the avatar's head movement toward the target, choosing the sign in each instance such that deviations of gaze from the straight ahead toward the target were positive and deviations to the opposite side were negative. To quantify whether the average trace deviated from 0, we used a pointwise *t*-test at each time point between 2500 ms before and after the peak head orientation, and we corrected for multiple comparisons by adjusting the alpha level to a 5% expected false-discovery rate (FDR) using the Benjamini and Hochberg (1995) procedure. We also compared the groups pointwise by using a two-sample *t*-test and applying the same correction of the alpha level to a 5% FDR.

#### Verbal responses of the participants

In addition to the analyses we preregistered, we also counted how many participants perceived the cue in the induction video as gaze based (e.g., as a helmet) and how many connected this head design with the cueing disk. This was based on verbal responses of the participants in the debriefing after the experiment. We used phi coefficients to quantify to what extent the perception of gaze allocation and the association of the helmet and the cueing disk were associated with the two groups. We treated the two groups as a binary variable.

## Results

### Gaze following during video presentation

**Figure 3.**
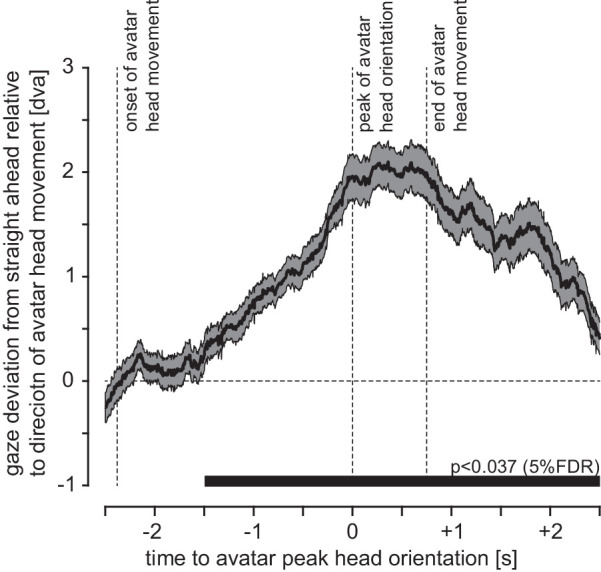
Deviation of horizontal gaze from the straight ahead in the direction of the avatar's head motion aligned to this motion in the induction videos. Shown are the mean and standard error of the mean across participants. The horizontal bar shows time points with a significant deviation from the straight-ahead (0) at an expected FDR of 5% (alpha = 0.037). The participant's gaze direction is given in degrees of visual angle (dva). For comparison, the peak head rotation of the avatar is 31.2° and reached 2 meters before the avatar crosses the target. At this point, the target appears at 20.2° for the participant, who follows the avatar at a constant distance of 1.29 meters throughout (see the Appendix for details regarding the geometry of the video).

Before testing the main hypothesis, we verified that the videos indeed induced gaze-following behavior. We found a significant deviation of participant's gaze from the straight-ahead in the direction of the avatar's head motion starting at 1.47 seconds prior to the peak orientation of the head (i.e., 0.91 seconds after the avatar began to move its head in the direction of the target) (Figure 3). Although there were sporadic differences between the two groups in this measure of horizontal deviation at an uncorrected 5% alpha level, the difference at none of these time points was significant at an alpha level adjusted to a 5% (or even a 10%) FDR.

### Cueing effects: Group differences and interaction

**Figure 4.**
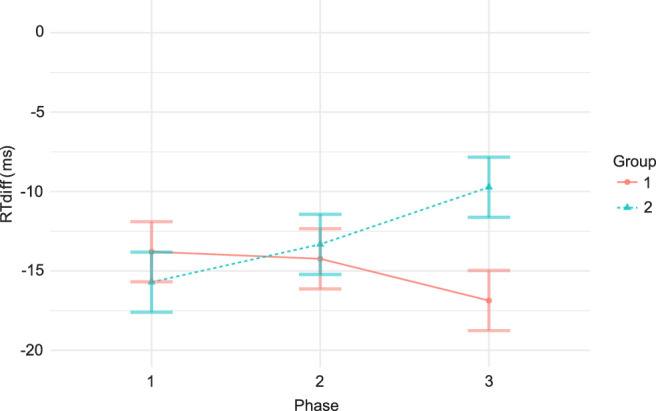
Differences of the median response times (RT_diff_) between feature-cued targets and gaze-cued targets.

We hypothesized a reversal (at least partial) of the cueing effect from a benefit for the feature-cued side to a benefit for the gaze-cued side after seeing the induction video. Because other factors may influence the development of the cueing effect over time, we formalized this hypothesis as a group difference in phase 2, where we expected the reaction time difference (gaze-cued trials minus feature-cued trials) to be smaller for group 1 than for group 2 (i.e., gaze-cued trials were, relatively speaking, becoming faster for group 1). To statistically account for the possibility that, despite the random assignment, there are already group differences in phase 1, we first computed a 2 × 3 repeated-measures ANOVA with group as the between-subject factor and experiment phase as the within-subject factor, with the dependent variable reaction time difference defined in the section Reaction time and accuracy. Effects of the induction video should manifest itself in a group × experiment phase interaction. There was neither a main effect of group, *F*(1, 40) = 0.65, *p* = 0.42, nor of experiment phase, *F*(2, 80) = 0.60, *p* = 0.55, but (as hypothesized) there was a significant interaction between the two factors, *F*(2, 80) = 5.92, *p* = 0.004 (Figure 4).

**Figure 5.**
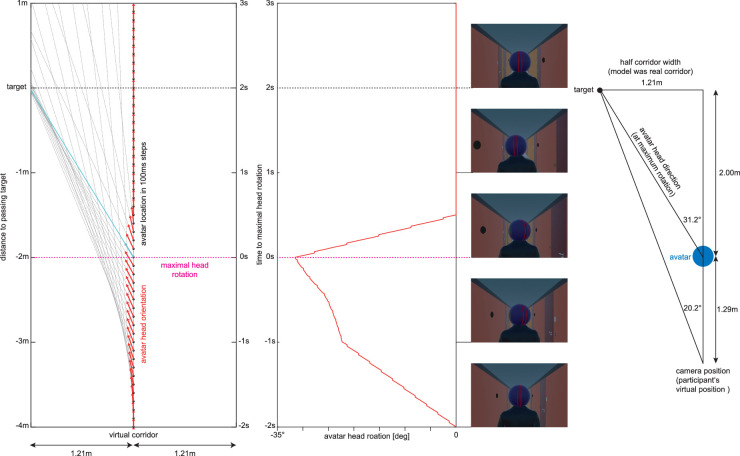
Avatar head movements when passing a target on the left wall. (Left) Position of the avatar relative to target (black spot on the left wall) in steps of 100 ms with the viewing direction (red arrow) and intersection between the viewing direction and side wall (dashed gray lines). The scale on the left gives the distance to the passage of the target (black dotted line); the scale on the right gives the time relative to the maximal head rotation, which occurs 2 meters (i.e., 2 seconds, as the speed is constant at 1 m/s) prior to passing the target (dotted magenta line). (Middle left) On the same vertical axis as the left panel, the head rotation is shown for the rotation to the left. (Middle right, from bottom to top) Video frames of the induction video at 2 seconds before the maximum head rotation (start of head rotation), 1 second before the maximum head rotation (head direction has reached target and continues to track), at maximum head rotation, and at 1 second and 2 seconds after the maximum head rotation (head is pointing straight ahead with some jitter due to the avatar's walking movement). (Right) Geometry with the participant on the same scale as the left panels. For targets on the right wall, movements are mirrored but otherwise identical.

As defined in our preregistration, we computed the group differences in each experiment phase by independent sample *t*-tests as follow-up on the significant interaction. In phase 1, we found no group difference, *M*_1_ = 13.79, *SD*_1_ = 7.01; *M*_2_ = 15.70, *SD*_2_ = 7.26; *t*(40) = 0.87, *p* = 0.39. This was to be expected, as both groups were treated identically in phase 1. In phase 2, we found no significant difference between the groups, either (*M*_1_ = 14.23, *SD*_1_ = 8.77; *M*_2_ = 13.32, *SD*_2_ = 10.76; *t*(40) = 0.30, *p* = 0.76). This was contrary to our hypothesis. In phase 3, we found a significantly smaller RT_diff_ for group 1 (*M*_1_ = –16.86, *SD*_1_ = 11.85) than for group 2 (*M*_2_ = –9.73, *SD*_2_ = 10.95), *t*(40) = –2.03, *p* = 0.049. Although at first glance these findings are inconsistent with our original hypothesis, the phase 2 results and the significant interaction indicate that the hypothesized effect of the partial reversal from feature cue to face cue occurred for group 2 in phase 3, although not for group 1 in phase 2.

### Verbal responses of the participants

Based on verbal statements of the participants in the debriefing after the experiment, we counted how many participants perceived the cue in the induction video as gaze related (e.g., as helmet) and how many associated this head design with the cuing disk without being prompted. All 21 participants in group 2 perceived that the avatar shifted its gaze in the induction video, whereas 16 in group 1 did so (phi = 0.37). There were also numerically more participants in group 2 (20) who spontaneously reported that they noticed the similarity of the “ball/ helmet” in the video and the disk compared with group 1 (15; phi = 0.32). According to the phi coefficient we found weak to moderate group correlations of the perception of gaze allocation and the association of the cueing stimulus with the helmet in the direction of group 2.

## Discussion

In the present study, we first showed that the motion of a cue toward one side of the display resulted in a robust attentional benefit on this side, even though the motion of the cue was not informative with regard to the location of the target. Interpreting the motion of the cue as a head movement toward the opposite side modulated this effect, but not as immediately and as robustly as hypothesized. This applied even though most participants were clearly aware of the interpretation of the cue as the back of a head (helmet) and reported so without being explicitly prompted, and the induction video clearly led to gaze-following behavior.

These results show that motion acts as a cue and provide evidence that attention is shifted in the direction of motion, even if the motion cues are not informative. This effect is consistent with findings that movements attract attention (e.g., abrupt onsets, Christ & Abrams, 2006, Yantis & Jonides, 1984; optic flow, Higuchi, Inoue, Endo, & Kumada, 2019) and that non-predictive shifts of vertical lines can act as attention cues (Gregory & Jackson, 2017). With the, on average (first phase), RT of 13 ms, the RT advantage induced by motion in our study was comparably large, given it did not come with a behavioral benefit and might even be detrimental to performance. This shows that motion cues cannot be ignored—similar to arrows or eyes (Galfano et al., 2012).

By presenting induction videos we tried to transform the feature-based cue into a social cue, with the social effect directed in the opposite direction from the feature-based effect. We therefore expected that the social interpretation would counteract the feature-based effect or even revert it. As hypothesized, we found a statistical interaction between group and experiment phase; however, we would have expected a pattern that more clearly showed a difference during the second phase. We expected that group 2 would retain its feature-cue advantage, whereas group 1 would shift toward a less feature-cue advantage or even a gaze-cue advantage. In fact, we observed no difference between the groups, and both clearly retained a feature-cue advantage. For phase 3, we had expected group 2 to shift more toward the gaze-cue advantage, whereas group 1 would retain its level or shift slightly back toward a more feature-cue advantage. Interestingly, the shift toward a more gaze-cue advantage in group 2 was observed (although the net benefit was still toward the feature-cued side), as was the shift toward a more feature-cue advantage for group 1, to an extent that a significant group difference is apparent in phase 3. Hence, there was an effect of the induction video compared to the control video, but it manifested itself only in phase 3. The role of the control video in reverting the effect may be stronger than expected, and in all cases the feature-cue advantage prevailed over the gaze-cue advantage.

We assume that these ambiguous results were caused by interacting mechanisms, each of which also had at least some effect on response times. First, the pure motion cue acted in all phases and groups. Second, the induction videos had an effect, but the effect was comparatively small and did not lead to a complete reversal of the cueing effect. This could probably be attributed to the fact that the first induction video was presented only after 300 cues had already been presented in the baseline phase (phase 1). Hence, the non-informativeness of the cue had been firmly established at this point, but—as the performance was near ceiling and there was neither explicit nor implicit feedback on reaction times—no “unlearning” of the motion-based effect should take place. In addition, it is plausible that a feature-based attentional shift occurs more quickly than one based on a social interpretation of the stimulus.

One might argue that presenting the eyes rather than the back of the head would have led to larger effects. Although we cannot exclude this based on the present data, head orientation clearly has an impact on estimated gaze direction, even if in conflict with the eye orientation. Participants are biased by head orientation when estimating gaze direction from pictures (Wollaston, 1824; for a review, see Langton, Watt, & Bruce, 2000). Moreover, in a real-life setting similar to our induction video, gaze following is larger for distancing pedestrians (seen from behind) than for oncoming pedestrians (seen from the front) (Gallup, Chong, & Couzin, 2012). Finally, there clearly was robust gaze following for the induction video, as participants directed their gaze to the walls following the avatar's gaze (Figure 3).

Despite the gaze-following behavior in the induction video and despite most participants being aware of the link between the avatar's helmet and the abstract cue, spontaneous reports of several participants after the experiment seemed to imply that their perception alternated between perceiving the blue disk as a head seen from behind and as a simple geometric cue. Judging solely from their (anecdotal) report, it is conceivable that they experienced some form of perceptual multistability (cf. Leopold & Logothetis, 1999). As the current perceptual interpretation of an ambiguous stimulus can impact a variety of other visual and attentional effects, such as object-based attention (Naber, Carlson, Verstraten, & Einhäuser, 2011) and the efficacy of a stimulus as a visual prime (Cave, Blake, & McNamara, 1998), it is likely that, in these cases, feature-based effects and gaze-based effects alternated, reducing gaze-based effects on average. Interestingly, in a situation in which gaze direction has to be actively inferred in each trial through mental attribution for a stimulus that is ambiguous at the time of target onset (two identical face masks pointing in opposite directions, where one could be attributed to gaze, the other to the back of the head), no gaze cuing effect was found (Kingstone, Kachkovski, Vasilyev, Kuk, & Welsh, 2019). For our present paradigm, this may indicate that, in our case, the attribution process (interpretation of the geometric stimulus as gaze), which occurs once prior to the block rather than in each trial, is strong enough to overcome such ambiguity on average, but only to a small extent. Our observation that prior induction of a stimulus as gaze can yield gaze-following effects is in line with 12-month-old infants exhibiting gaze-following behavior after observing a “social” interaction with a faceless object (Johnson, Slaughter, & Carey, 1998). Although this object is initially meaningless in terms of attention direction, in our case the interpretation of the cue as gaze had to overcome the opposing attentional effect of the motion of the cue.

One reason why significant differences did not appear until the third phase may be the contrast effects between the control and induction videos. The absence of any gaze behavior by the avatar in the control video in phase 2 may have led to an increased impression of gaze alignment in group 2 in phase 3. For group 1, on the other hand, the absence of gaze behavior may have led to a slight emphasis on the feature cue. For gaze direction perception, it has already been shown that one stimulus can influence the perception of another stimulus even after minutes (Kloth et al., 2017). Although these effects did not lead to significant differences between phases within groups, they may have caused the statistical interaction. This assumption is supported by the fact that slightly more participants in group 2 reported perception of gaze allocation and an association of the cueing stimulus with the helmet.

We deliberately chose a design in which the cue was not informative; that is, the cue was valid and invalid in an equal amount of trials, which is different from the paradigm of Posner (1980), where the effect of the cue is a consequence of its validity. Instead, the usage of the cue is more similar to cueing studies that use special cues, such as eyes (Friesen & Kingstone, 1998) or eyes and arrows (Galfano et al., 2012; Ristic et al., 2007). As a consequence, the social cue had to counter the feature cue with no evident behavioral cost or benefit, which might diminish potential effects. It will be an interesting approach for further research to determine whether combining the cue with different levels of validity, such that in some cases the information is in conflict with the present effect, will lead to different trade-offs depending on interpretation of the cue as a head. We also deliberately chose a simple reaction task, where participants responded congruently (left hand for left, right hand for right) to the side of target appearance. This might further benefit the motion cue, as in valid trials it cues not only the side of the target appearance but also the side of the response, whereas the gaze cue might be perceived as limited to a narrower region on the screen. It also makes the task rather easy to perform, such that cueing effects can only manifest themselves in reaction times but not in performance differences. The use of an identification task might have also been conceivable (as was done for gaze cueing in Friesen & Kingstone, 1998); however, it might add a further complication due to the congruent or incongruent response mapping, which is why we refrained from using this approach in our study.

Our videos tried to transform an abstract geometric figure devoid of any semantic content to a social cue without any behavioral benefit. In contrast, under real-world conditions, potential cues prove useful and are used when they are reliable. In this study, however, we showed that social cues are used, even if they have initially no resemblance to known social cue appearances, such as eyes or hand gestures, and come with no evident behavioral benefit. Future research could investigate the transformation of contentless features into social cues in more complex and realistic scenarios. The presentation of videos from a virtual reality for induction purposes, as we have done here, is likely only a first step. Further research could involve linking cues to manual actions by artificial agents, which has received little attention so far when considering cueing and visual attention (Atkinson, Simpson, & Cole, 2018). Furthermore, it would be interesting to investigate whether a cue that represents visual attentional orienting of others can also lead to differences in time perception (Conty et al., 2016). This would help to distinguish whether transforming an abstract into a social cue represents a simple learning of a (presumed) cue–target association or whether after induction the cue actually is perceived as social. In either case, our present data clearly show that the reinterpretation of a geometric figure as social stimulus (gaze direction) can impact its efficacy as an attentional cue.

## Supplementary Material

Supplement 1

Supplement 2

Supplement 3

Supplement 4

## Appendix

The avatar's head was designed such that he moves his head smoothly toward the target starting about 4 meters before the target is reached (Figure 5). He reaches a maximum head turn of 31.2° at a distance of 2.00 meters before the target and then turns his head back. The participant (virtual camera) follows the avatar at a fixed (virtual) distance of 1.29 meters. From the perspective of the participant, the target can appear at practically every angle (it is at 90° at the point where the target is passed), but at the time of the avatar's maximal head turn it is at 20.2° relative to the straight ahead (Figure 5, right).

## Supplementary material

Supplementary Movie S1. Induction video.

Supplementary Movie S2. Mirrored induction video.

Supplementary Movie S3. Control video.

Supplementary Movie S4. Mirrored control video.
